# Supplementary material for: Iterative improvement in the automatic modular design of robot swarms
Source: PeerJ Comput Sci. 2020 Dec 7;6:e322. doi: 10.7717/peerj-cs.322 (PMC7924708; doi:10.7717/peerj-cs.322)
Supplement: Supplemental Information 3 [file peerj-cs-06-322-s003.zip › argos3/doc/api/standalone/a00658_source.html]

ARGoS: plugins/simulator/visualizations/qt-opengl/qtopengl\_user\_functions.cpp Source File


- Main Page
- Related Pages
- Namespaces
- Classes
- Files

- File List
- File Members

# plugins/simulator/visualizations/qt-opengl/qtopengl\_user\_functions.cpp

Go to the documentation of this file.

```
00001 
00008 #include "qtopengl_user_functions.h"
00009 #include <QPainter>
00010 
00011 namespace argos {
00012 
00013    /****************************************/
00014    /****************************************/
00015 
00016    const GLfloat DEFAULT_SPECULAR[]  = {   0.0f, 0.0f, 0.0f, 1.0f };
00017    const GLfloat DEFAULT_SHININESS[] = { 100.0f                   };
00018    const GLfloat DEFAULT_EMISSION[]  = {   0.0f, 0.0f, 0.0f, 1.0f };
00019 
00020    /****************************************/
00021    /****************************************/
00022 
00023    static void Rototranslate(const CVector3& c_position,
00024                              const CQuaternion& c_orientation) {
00025       /* Get Euler angles */
00026       CRadians cZAngle, cYAngle, cXAngle;
00027       c_orientation.ToEulerAngles(cZAngle, cYAngle, cXAngle);
00028       /* Translate */
00029       glTranslatef(c_position.GetX(), c_position.GetY(), c_position.GetZ());
00030       /* Rotate */
00031       glRotatef(ToDegrees(cXAngle).GetValue(), 1.0f, 0.0f, 0.0f);
00032       glRotatef(ToDegrees(cYAngle).GetValue(), 0.0f, 1.0f, 0.0f);
00033       glRotatef(ToDegrees(cZAngle).GetValue(), 0.0f, 0.0f, 1.0f);
00034    }
00035 
00036    /****************************************/
00037    /****************************************/
00038 
00039    CQTOpenGLUserFunctions::CQTOpenGLUserFunctions() :
00040       m_vecFunctionHolders(1),
00041       m_pcQTOpenGLMainWindow(NULL) {
00042       m_cThunks.Add<CEntity>((TThunk)NULL);
00043    }
00044 
00045    /****************************************/
00046    /****************************************/
00047 
00048    CQTOpenGLUserFunctions::~CQTOpenGLUserFunctions() {
00049       while(!m_vecFunctionHolders.empty()) {
00050          delete m_vecFunctionHolders.back();
00051          m_vecFunctionHolders.pop_back();
00052       }
00053    }
00054 
00055    /****************************************/
00056    /****************************************/
00057 
00058    void CQTOpenGLUserFunctions::KeyPressed(QKeyEvent* pc_event) {
00059       m_pcQTOpenGLMainWindow->GetOpenGLWidget().KeyPressed(pc_event);
00060    }
00061 
00062    /****************************************/
00063    /****************************************/
00064 
00065    void CQTOpenGLUserFunctions::KeyReleased(QKeyEvent* pc_event) {
00066       m_pcQTOpenGLMainWindow->GetOpenGLWidget().KeyReleased(pc_event);
00067    }
00068    
00069    /****************************************/
00070    /****************************************/
00071 
00072    CEntity* CQTOpenGLUserFunctions::GetSelectedEntity() {
00073       return m_pcQTOpenGLMainWindow->GetOpenGLWidget().GetSelectedEntity();
00074    }
00075 
00076    /****************************************/
00077    /****************************************/
00078 
00079    void CQTOpenGLUserFunctions::SelectEntity(CEntity& c_entity) {
00080       m_pcQTOpenGLMainWindow->GetOpenGLWidget().SelectEntity(c_entity);
00081    }
00082 
00083    /****************************************/
00084    /****************************************/
00085 
00086    void CQTOpenGLUserFunctions::DeselectEntity() {
00087       m_pcQTOpenGLMainWindow->GetOpenGLWidget().DeselectEntity();
00088    }
00089 
00090    /****************************************/
00091    /****************************************/
00092 
00093    CQTOpenGLMainWindow& CQTOpenGLUserFunctions::GetMainWindow() {
00094       return *m_pcQTOpenGLMainWindow;
00095    }
00096 
00097    /****************************************/
00098    /****************************************/
00099 
00100    void CQTOpenGLUserFunctions::SetMainWindow(CQTOpenGLMainWindow& c_main_win) {
00101       m_pcQTOpenGLMainWindow = &c_main_win;
00102    }
00103 
00104    /****************************************/
00105    /****************************************/
00106 
00107    CQTOpenGLWidget& CQTOpenGLUserFunctions::GetQTOpenGLWidget() {
00108       return m_pcQTOpenGLMainWindow->GetOpenGLWidget();
00109    }
00110 
00111    /****************************************/
00112    /****************************************/
00113 
00114    void CQTOpenGLUserFunctions::SetColor(const CColor& c_color) {
00115       const GLfloat pfColor[]     = {
00116          c_color.GetRed()   / 255.0f,
00117          c_color.GetGreen() / 255.0f,
00118          c_color.GetBlue()  / 255.0f
00119       };
00120       glMaterialfv(GL_FRONT_AND_BACK, GL_SPECULAR, DEFAULT_SPECULAR);
00121       glMaterialfv(GL_FRONT_AND_BACK, GL_SHININESS, DEFAULT_SHININESS);
00122       glMaterialfv(GL_FRONT_AND_BACK, GL_EMISSION, DEFAULT_EMISSION);
00123       glMaterialfv(GL_FRONT_AND_BACK, GL_AMBIENT_AND_DIFFUSE, pfColor);
00124    }
00125 
00126    /****************************************/
00127    /****************************************/
00128 
00129    void CQTOpenGLUserFunctions::DrawPoint(const CVector3& c_position,
00130                                           const CColor& c_color,
00131                                           const Real f_diameter) {
00132       /* Save attributes */
00133       glPushAttrib(GL_POINT_BIT);
00134       /* Set color */
00135       SetColor(c_color);
00136       /* Set point attributes */
00137       glPointSize(f_diameter);
00138       glEnable(GL_POINT_SMOOTH);
00139       /* Draw */
00140       glBegin(GL_POINTS);
00141       glVertex3f(c_position.GetX(), c_position.GetY(), c_position.GetZ());
00142       glEnd();
00143       /* Restore saved attributes */
00144       glPopAttrib();
00145    }
00146 
00147    /****************************************/
00148    /****************************************/
00149 
00150    void CQTOpenGLUserFunctions::DrawTriangle(const CVector3& c_position,
00151                                              const CQuaternion& c_orientation,
00152                                              Real f_base,
00153                                              Real f_height,
00154                                              const CColor& c_color,
00155                                              const bool b_fill) {
00156       /* Save attributes and current matrix */
00157       glPushAttrib(GL_POLYGON_BIT);
00158       glPushMatrix();
00159       /* Set color */
00160       SetColor(c_color);
00161       /* Disable face culling, to make the triangle visible from any angle */
00162             glDisable(GL_CULL_FACE);
00163       /* Set polygon attributes */
00164             glEnable(GL_POLYGON_SMOOTH);
00165       glPolygonMode(GL_FRONT_AND_BACK, b_fill ? GL_FILL : GL_LINE);
00166       /* Set position/orientation */
00167       Rototranslate(c_position, c_orientation);
00168       /*  Draw */
00169       glBegin(GL_TRIANGLES);
00170       glNormal3f(0.0f, 0.0f, 1.0f);
00171       glVertex3f(-f_base * 0.5f,     0.0f, 0.0f);
00172       glVertex3f( f_base * 0.5f,     0.0f, 0.0f);
00173       glVertex3f(          0.0f, f_height, 0.0f);
00174       glEnd();
00175       /* Restore saved stuff */
00176       glPopMatrix();
00177       glPopAttrib();
00178    }
00179 
00180    /****************************************/
00181    /****************************************/
00182 
00183    void CQTOpenGLUserFunctions::DrawPolygon(const CVector3& c_position,
00184                                             const CQuaternion& c_orientation,
00185                                             const std::vector<CVector2>& vec_points,
00186                                             const CColor& c_color,
00187                                             const bool b_fill) {
00188       if(vec_points.size() < 2) {
00189          LOGERR << "CQTOpenGLUserFunctions::DrawPolygon() needs at least 3 points." << std::endl;
00190          return;
00191       }
00192       /* Save attributes and current matrix */
00193       glPushAttrib(GL_POLYGON_BIT);
00194       /* Set color */
00195       SetColor(c_color);
00196       /* Disable face culling, to make the triangle visible from any angle */
00197             glDisable(GL_CULL_FACE);
00198       /* Set polygon attributes */
00199             glEnable(GL_POLYGON_SMOOTH);
00200       glPolygonMode(GL_FRONT_AND_BACK, b_fill ? GL_FILL : GL_LINE);
00201       /* Set position/orientation */
00202       Rototranslate(c_position, c_orientation);
00203       /*  Draw */
00204       glBegin(GL_POLYGON);
00205       glNormal3f(0.0f, 0.0f, 1.0f);
00206       for(size_t i = 0; i < vec_points.size(); ++i) {
00207          glVertex3f(vec_points[i].GetX(), vec_points[i].GetY(), 0.0f);
00208       }
00209       glEnd();
00210       /* Reset translation */
00211       glTranslatef(-c_position.GetX(), -c_position.GetY(), -c_position.GetZ());
00212       /* Restore saved stuff */
00213       glPopAttrib();
00214    }
00215 
00216    /****************************************/
00217    /****************************************/
00218 
00219    void CQTOpenGLUserFunctions::DrawCircle(const CVector3& c_position,
00220                                            const CQuaternion& c_orientation,
00221                                            Real f_radius,
00222                                            const CColor& c_color,
00223                                            const bool b_fill,
00224                                            GLuint un_vertices) {
00225       /* Save attributes and current matrix */
00226       glPushAttrib(GL_POLYGON_BIT);
00227       glPushMatrix();
00228       /* Set color */
00229       SetColor(c_color);
00230       /* Disable face culling, to make the triangle visible from any angle */
00231             glDisable(GL_CULL_FACE);
00232       /* Set polygon attributes */
00233             glEnable(GL_POLYGON_SMOOTH);
00234       glPolygonMode(GL_FRONT_AND_BACK, b_fill ? GL_FILL : GL_LINE);
00235       /* Set position/orientation */
00236       Rototranslate(c_position, c_orientation);
00237       /*  Draw */
00238             CVector2 cVertex(f_radius, 0.0f);
00239             CRadians cAngle(CRadians::TWO_PI / un_vertices);
00240       glBegin(GL_POLYGON);
00241       glNormal3f(0.0f, 0.0f, 1.0f);
00242       for(size_t i = 0; i < un_vertices; ++i) {
00243          glVertex3f(cVertex.GetX(), cVertex.GetY(), 0.0f);
00244          cVertex.Rotate(cAngle);
00245       }
00246       glEnd();
00247       /* Restore saved stuff */
00248       glPopAttrib();
00249       glPopMatrix();
00250    }
00251 
00252    /****************************************/
00253    /****************************************/
00254 
00255    void CQTOpenGLUserFunctions::DrawCylinder(const CVector3& c_position,
00256                                              const CQuaternion& c_orientation,
00257                                              Real f_radius,
00258                                              Real f_height,
00259                                              const CColor& c_color,
00260                                              GLuint un_vertices) {
00261       /* Save current matrix */
00262       glPushMatrix();
00263       /* Set color */
00264       SetColor(c_color);
00265       /* Set position/orientation */
00266       Rototranslate(c_position, c_orientation);
00267       /* Draw side surface */
00268       Real fHalfHeight = f_height * 0.5f;
00269       CVector2 cVertex(1.0f, 0.0f);
00270       CRadians cAngle(CRadians::TWO_PI / un_vertices);
00271       glBegin(GL_QUAD_STRIP);
00272       for(GLuint i = 0; i <= un_vertices; i++) {
00273          glNormal3f(cVertex.GetX(), cVertex.GetY(), 0.0f);
00274          glVertex3f(cVertex.GetX() * f_radius, cVertex.GetY() * f_radius,  fHalfHeight);
00275          glVertex3f(cVertex.GetX() * f_radius, cVertex.GetY() * f_radius, -fHalfHeight);
00276          cVertex.Rotate(cAngle);
00277       }
00278       glEnd();
00279       /* Draw top disk */
00280       cVertex.Set(f_radius, 0.0f);
00281       glBegin(GL_POLYGON);
00282       glNormal3f(0.0f, 0.0f, 1.0f);
00283       for(GLuint i = 0; i <= un_vertices; i++) {
00284          glVertex3f(cVertex.GetX(), cVertex.GetY(), fHalfHeight);
00285          cVertex.Rotate(cAngle);
00286       }
00287       glEnd();
00288       /* Draw bottom disk */
00289       cVertex.Set(f_radius, 0.0f);
00290       cAngle = -cAngle;
00291       glBegin(GL_POLYGON);
00292       glNormal3f(0.0f, 0.0f, -1.0f);
00293       for(GLuint i = 0; i <= un_vertices; i++) {
00294          glVertex3f(cVertex.GetX(), cVertex.GetY(), -fHalfHeight);
00295          cVertex.Rotate(cAngle);
00296       }
00297       glEnd();
00298       /* Restore saved matrix */
00299       glPopMatrix();
00300    }
00301 
00302    /****************************************/
00303    /****************************************/
00304 
00305    void CQTOpenGLUserFunctions::DrawBox(const CVector3& c_position,
00306                                         const CQuaternion& c_orientation,
00307                                         const CVector3& c_size,
00308                                         const CColor& c_color) {
00309       /* Save current matrix */
00310       glPushMatrix();
00311       /* Set color */
00312       SetColor(c_color);
00313       /* Set position/orientation */
00314       Rototranslate(c_position, c_orientation);
00315       /* Draw top and bottom faces (parallel to XY) */
00316       CVector3 cHalfSize = c_size * 0.5f;
00317       glBegin(GL_QUADS);
00318       /* Bottom face */
00319       glNormal3f(0.0f, 0.0f, -1.0f);
00320       glVertex3f( cHalfSize.GetX(),  cHalfSize.GetY(), -cHalfSize.GetZ());
00321       glVertex3f( cHalfSize.GetX(), -cHalfSize.GetY(), -cHalfSize.GetZ());
00322       glVertex3f(-cHalfSize.GetX(), -cHalfSize.GetY(), -cHalfSize.GetZ());
00323       glVertex3f(-cHalfSize.GetX(),  cHalfSize.GetY(), -cHalfSize.GetZ());
00324       /* Top face */
00325       glNormal3f(0.0f, 0.0f, 1.0f);
00326       glVertex3f(-cHalfSize.GetX(), -cHalfSize.GetY(),  cHalfSize.GetZ());
00327       glVertex3f( cHalfSize.GetX(), -cHalfSize.GetY(),  cHalfSize.GetZ());
00328       glVertex3f( cHalfSize.GetX(),  cHalfSize.GetY(),  cHalfSize.GetZ());
00329       glVertex3f(-cHalfSize.GetX(),  cHalfSize.GetY(),  cHalfSize.GetZ());
00330       glEnd();
00331       /* Draw the other faces (South, East, North, West) */
00332       glBegin(GL_QUADS);
00333       /* North face */
00334       glNormal3f(1.0f, 0.0f, 0.0f);
00335       glVertex3f( cHalfSize.GetX(), -cHalfSize.GetY(), -cHalfSize.GetZ());
00336       glVertex3f( cHalfSize.GetX(),  cHalfSize.GetY(), -cHalfSize.GetZ());
00337       glVertex3f( cHalfSize.GetX(),  cHalfSize.GetY(),  cHalfSize.GetZ());
00338       glVertex3f( cHalfSize.GetX(), -cHalfSize.GetY(),  cHalfSize.GetZ());
00339       /* South face */
00340       glNormal3f(-1.0f, 0.0f, 0.0f);
00341       glVertex3f(-cHalfSize.GetX(), -cHalfSize.GetY(), -cHalfSize.GetZ());
00342       glVertex3f(-cHalfSize.GetX(), -cHalfSize.GetY(),  cHalfSize.GetZ());
00343       glVertex3f(-cHalfSize.GetX(),  cHalfSize.GetY(),  cHalfSize.GetZ());
00344       glVertex3f(-cHalfSize.GetX(),  cHalfSize.GetY(), -cHalfSize.GetZ());
00345       /* East face */
00346       glNormal3f(0.0f, -1.0f, 0.0f);
00347       glVertex3f(-cHalfSize.GetX(), -cHalfSize.GetY(), -cHalfSize.GetZ());
00348       glVertex3f( cHalfSize.GetX(), -cHalfSize.GetY(), -cHalfSize.GetZ());
00349       glVertex3f( cHalfSize.GetX(), -cHalfSize.GetY(),  cHalfSize.GetZ());
00350       glVertex3f(-cHalfSize.GetX(), -cHalfSize.GetY(),  cHalfSize.GetZ());
00351       /* West face */
00352       glNormal3f(0.0f, 1.0f, 0.0f);
00353       glVertex3f(-cHalfSize.GetX(),  cHalfSize.GetY(), -cHalfSize.GetZ());
00354       glVertex3f(-cHalfSize.GetX(),  cHalfSize.GetY(),  cHalfSize.GetZ());
00355       glVertex3f( cHalfSize.GetX(),  cHalfSize.GetY(),  cHalfSize.GetZ());
00356       glVertex3f( cHalfSize.GetX(),  cHalfSize.GetY(), -cHalfSize.GetZ());
00357       glEnd();
00358       /* Restore saved matrix */
00359       glPopMatrix();
00360    }
00361 
00362    /****************************************/
00363    /****************************************/
00364 
00365    void CQTOpenGLUserFunctions::DrawRay(const CRay3& c_ray,
00366                                         const CColor& c_color,
00367                                         Real f_width) {
00368       /* Save attributes and current matrix */
00369       glPushAttrib(GL_LINE_BIT | GL_ENABLE_BIT);
00370       /* Set line attributes */
00371       glEnable(GL_LINE_SMOOTH);
00372       glLineWidth(f_width);
00373       /* Unset lighting */
00374       glDisable(GL_LIGHTING);
00375       glDisable(GL_COLOR_MATERIAL);
00376       /* Set color */
00377       glColor3ub(c_color.GetRed(), c_color.GetGreen(), c_color.GetBlue());
00378       /* Draw ray */
00379       glBegin(GL_LINES);
00380       glVertex3f(c_ray.GetStart().GetX(), c_ray.GetStart().GetY(), c_ray.GetStart().GetZ());
00381       glVertex3f(c_ray.GetEnd().GetX(), c_ray.GetEnd().GetY(), c_ray.GetEnd().GetZ());
00382       glEnd();
00383       /* Restore saved stuff */
00384       glPopAttrib();
00385    }
00386 
00387    /****************************************/
00388    /****************************************/
00389 
00390    static void TransformPoint(Real* pf_vec_out,
00391                               const Real* pf_trans,
00392                               const Real* pf_vec_in) {
00393 #define M(row,col) pf_trans[col * 4 + row]
00394       pf_vec_out[0] =
00395          M(0, 0) * pf_vec_in[0] +
00396          M(0, 1) * pf_vec_in[1] +
00397          M(0, 2) * pf_vec_in[2] +
00398          M(0, 3) * pf_vec_in[3];
00399       pf_vec_out[1] =
00400          M(1, 0) * pf_vec_in[0] +
00401          M(1, 1) * pf_vec_in[1] +
00402          M(1, 2) * pf_vec_in[2] +
00403          M(1, 3) * pf_vec_in[3];
00404       pf_vec_out[2] =
00405          M(2, 0) * pf_vec_in[0] +
00406          M(2, 1) * pf_vec_in[1] +
00407          M(2, 2) * pf_vec_in[2] +
00408          M(2, 3) * pf_vec_in[3];
00409       pf_vec_out[3] =
00410          M(3, 0) * pf_vec_in[0] +
00411          M(3, 1) * pf_vec_in[1] +
00412          M(3, 2) * pf_vec_in[2] +
00413          M(3, 3) * pf_vec_in[3];
00414 #undef M
00415    }
00416 
00417    /****************************************/
00418    /****************************************/
00419 
00420    void CQTOpenGLUserFunctions::DrawText(const CVector3& c_position,
00421                                          const std::string& str_text,
00422                                          const CColor& c_color,
00423                                          const QFont& c_font) {
00424       /* Get current modelview matrix */
00425       GLdouble pf_mv[16];
00426       glGetDoublev(GL_MODELVIEW_MATRIX, pf_mv);
00427       /* Get current projection matrix */
00428       GLdouble pf_proj[16];
00429       glGetDoublev(GL_PROJECTION_MATRIX, pf_proj);
00430       /* Get current viewport */
00431       GLint pn_vp[4];
00432       glGetIntegerv(GL_VIEWPORT, pn_vp);
00433       /* Buffers for projection calculations */
00434       GLdouble pf_v1[4], pf_v2[4];
00435       /* Transform original position with modelview matrix  */
00436       pf_v1[0] = c_position.GetX();
00437       pf_v1[1] = c_position.GetY();
00438       pf_v1[2] = c_position.GetZ();
00439       pf_v1[3] = 1.0;
00440       TransformPoint(pf_v2, pf_mv, pf_v1);
00441       /* Transform pf_v2 with projection matrix */
00442       TransformPoint(pf_v1, pf_proj, pf_v2);
00443       /* Failed projection? Get out */
00444       if (pf_v1[3] == 0.0) return;
00445       /* Rescale projection */
00446       pf_v1[0] /= pf_v1[3];
00447       pf_v1[1] /= pf_v1[3];
00448       /* Get screen position (before mirroring) */
00449       pf_v2[0] =
00450          (pn_vp[0] + (1. + pf_v1[0]) * pn_vp[2] / 2.) /
00451          GetMainWindow().devicePixelRatio();
00452       pf_v2[1] =
00453          (pn_vp[1] + (1. + pf_v1[1]) * pn_vp[3] / 2.) /
00454          GetMainWindow().devicePixelRatio();
00455       /* Mirror along Y because screen goes up to down */
00456       pf_v2[1] = GetMainWindow().GetOpenGLWidget().height() - pf_v2[1];
00457       /* Save OpenGL attributes */
00458       glPushAttrib(GL_ENABLE_BIT);
00459       /* Disable lighting to make text color unaffected by light */
00460       glDisable(GL_LIGHTING);
00461       glDisable(GL_COLOR_MATERIAL);
00462       /* Disable culling to make text visibile from any angle */
00463       glDisable(GL_CULL_FACE);
00464       /* Initialize Qt screen painter */
00465       QPainter cPainter(&GetMainWindow().GetOpenGLWidget());
00466       /* Set antialiasing */
00467       cPainter.setRenderHints(QPainter::Antialiasing | QPainter::TextAntialiasing);
00468       /* Set pen color */
00469       cPainter.setPen(
00470          QColor(c_color.GetRed(),
00471                 c_color.GetGreen(),
00472                 c_color.GetBlue(),
00473                 c_color.GetAlpha()));
00474       /* Set font */
00475       cPainter.setFont(c_font);
00476       /* Render text */
00477       cPainter.drawText(pf_v2[0], pf_v2[1], str_text.c_str());
00478       /* Dispose of Qt screen painter */
00479       cPainter.end();
00480       /* Restore saved attributes */
00481       glPopAttrib();
00482    }
00483 
00484    /****************************************/
00485    /****************************************/
00486 
00487    void CQTOpenGLUserFunctions::Call(CEntity& c_entity) {
00488       TThunk t_thunk = m_cThunks[c_entity.GetTag()];
00489       if(t_thunk) (this->*t_thunk)(c_entity);
00490    }
00491 
00492    /****************************************/
00493    /****************************************/
00494 
00495 }
```

---

Generated on 10 Jul 2018 for ARGoS by 
 1.6.1
